# Diagnosis of the Trigeminal Nerve Injury in a Patient with Pontine Hemorrhage

**DOI:** 10.3390/diagnostics10020074

**Published:** 2020-01-29

**Authors:** Eun Bi Choi, Jeong Pyo Seo, Sung Ho Jang

**Affiliations:** 1 Department of Physical Medicine and Rehabilitation, College of Medicine, Yeungnam University, 317-1 Daemyungdong, Namku, Daegu 705-717, Korea; ceb0808@hanmail.net; 2 Department of Physical Therapy, College of Health Sciences, Dankook University, Cheonan 31116, Korea; raphael0905@hanmail.net

**Keywords:** trigeminal nerve, diffusion tensor imaging, diffusion tensor tractography, pontine hemorrhage, nerve injury

## Abstract

Herein, we present a patient who was diagnosed with trigeminal nerve injury following a pontine hemorrhage. A 38-year-old male was diagnosed with a left pontine hemorrhage and underwent conservative management at the neurosurgery department of a university hospital. After hemorrhage onset, he felt facial pain on the right side. After seven years, he visited the rehabilitation department of another hospital for evaluation of his right facial pain. He complained of somatosensory impairment and facial pain (tingling and cold sensation) on the right side as well as difficulty chewing and gait disturbance. On neurological examination, decreased touch sensation (approximately 30%) was observed on the right side of the face, in the oral cavity, and on the tongue (anterior two-thirds) as well as weakness of the right-sided masseter muscles. He also exhibitedallodynia without dysesthesia on the right side of the face. Diffusion tensor tractography showed the right trigeminal nerve to be discontinued at the anterior margin of the pons (arrow) compared to the state of the left trigeminal nerve.

##  

**Figure 1:**
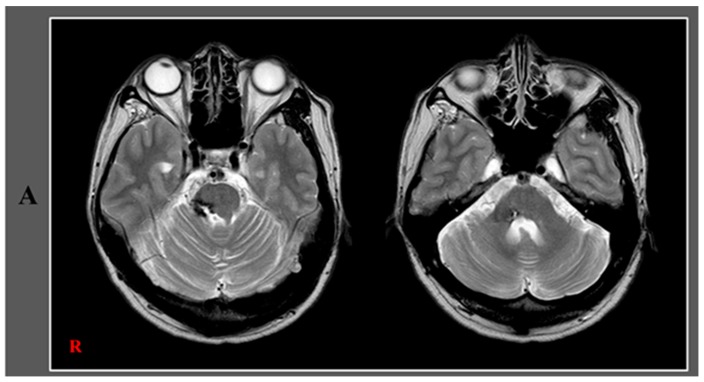

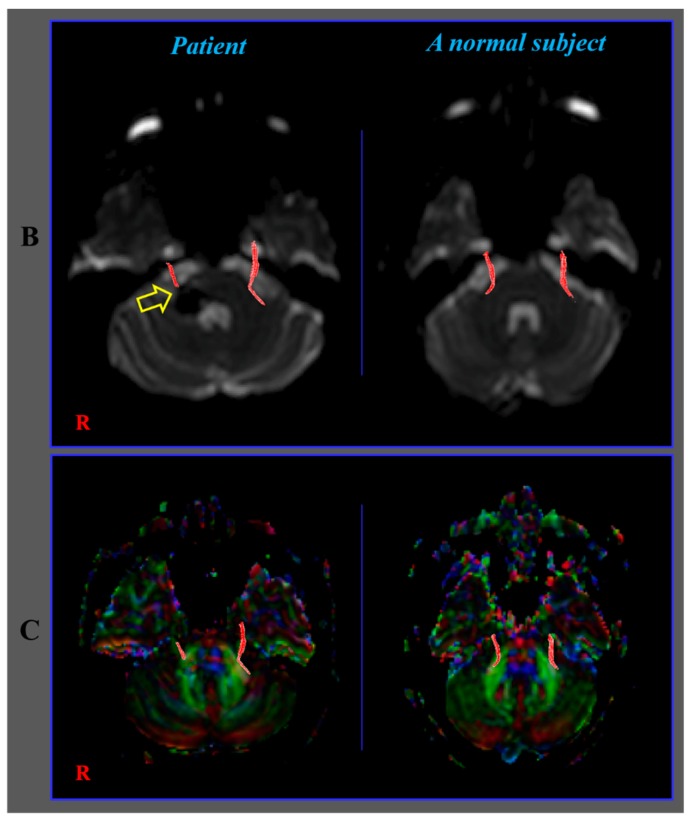
A 38-year-old male, diagnosed to havea left pontine hemorrhage, underwent conservative management at the neurosurgery department of a university hospital. After hemorrhage onset, he felt facial pain on the right side. Seven years later, he visited the rehabilitation department of another hospital for evaluation of his right-sided facial pain. He complained of somatosensory impairment and facial pain (tingling and cold sensation) on the right side, difficulty chewing, and gait disturbance. On neurological examination, decreased touch sensation (approximately 30%) was observed on the right side of the face as well as in the oral cavity and on the tongue (anterior two-thirds). In addition, there was a weakness of the right-sided masseter muscles, intentional tremor, and dysmetria of the right hand, a wide-based gait due to a balance problem, and truncal ataxia. He also exhibited allodynia without dysesthesia on the right side of the face. A leukomalactic lesion located in the tegmentum of the right pons and the middle cerebellar peduncle was observed on brain Megnetic Resonance Imaging (**A**). The patient provided written informed consent and the study protocol was approved by the Institutional Research Board of Yeungnam university hospital (approval No. YUMC-2019-06-032) on 21 June 2019.Diffusion tensor imaging (DTI) data were acquired over an approximatefive minute periodat seven years after onset by using a sensitivity-encoding head coil on a 1.5-T Philips GyroscanIntera (Hoffman-LaRoche, Ltd, Best, The Netherlands) with single-shot echo-planar imaging and a navigator echo. For each of the 32 non-collinear diffusion sensitizing gradients, 67 contiguous slices (acquisition matrix = 96 × 96; reconstructed to matrix = 192 × 192 matrix; field of view = 240 mm × 240 mm; repetition time = 10,398 ms; echo time = 72 ms; parallel imaging reduction factor (SENSE factor) = 2; Echo-planar imaging factor = 59; *b* = 1000 s/mm^2^; number of excitations = 1; and slice thickness = 2.5 mm) were acquired parallel to the anterior commissure–posterior commissure line. Eddy current-induced image distortions were removed by using affine multi-scale two-dimensional registration as provided in software from the Oxford Center for the Functional Magnetic Resonance Imaging of the Brain Software Library (www.fmrib.ox.ac.uk/fsl) [1]. DTI-Studio software (CMRM, Johns Hopkins Medical Institute, Baltimore, MD, USA; deterministic tractography) was used for the evaluation of the trigeminal nerve. The seed region of interest (ROI) was placed on the prepontine cistern and the target ROI was placed on isolated distal branches (seed ROI size: 20 voxels, target ROI size: 4 voxels). Fiber tracking was performed using a fractional anisotropy (FA)threshold of >0.1 and a direction threshold <70° [2]. It took approximately ten minutes for the reconstruction of the trigeminal nerve. The right trigeminal nerve was shown to be discontinued at the anterior margin of the pons (arrow) compared with the left trigeminal nerve (**B**) and there were similar indications on the DTIcolor map (**C**). Moreover, the FA (0.26) and fiber number (41) values of the right trigeminal nerve were lower than those (FA: 0.32, fiber number: 53) of the left trigeminal nerve. There were no hemisphere-related differences in the apparent diffusion coefficient values (right: 0.53, and left: 0.53). The results of this study show injury of the subject’s right trigeminal nerve. The neurological manifestations related to the trigeminal nerve injury including somatosensory impairment of the right face, oral cavity, and tongue, neuropathic pain of the right face, and weakness of right masseter muscles coincided with the diffusion tensor tractography (DTT) images of a right trigeminal nerve injury [3]. Since the introduction of DTI, many studies have used DTI to demonstrate lesions of the trigeminal nerve, particularly trigeminal neuralgia [4,5,6,7]. However, to the best of our knowledge, this is the first case report that used DTT to aid in the diagnosis of a trigeminal nerve injury in a patient with a pontine hemorrhage. We suggest that DTT can be a useful tool in the detection of lesions of the trigeminal nerve following pontine hemorrhage. However, this study was limited because DTI was scanned at the chronic stage (seven years after onset) of hemorrhage. Therefore, prospective studies where DTIs are scanned at an early stage of hemorrhage could have prognostic value by the detection of the presence and severity of early nerve injury.
